# Supplementary material for: Single-Cell Transcriptomics Reveals the Complexity of the Tumor Microenvironment of Treatment-Naive Osteosarcoma
Source: Front Oncol. 2021 Jul 21;11:709210. doi: 10.3389/fonc.2021.709210 (PMC8335545; doi:10.3389/fonc.2021.709210)
Supplement: Supplementary Document 1 — Kaplan-Meier curve of the 518 genes associated with survival. [file DataSheet_1.zip › Supplementary Document 1/sur.PGBD5.pdf]

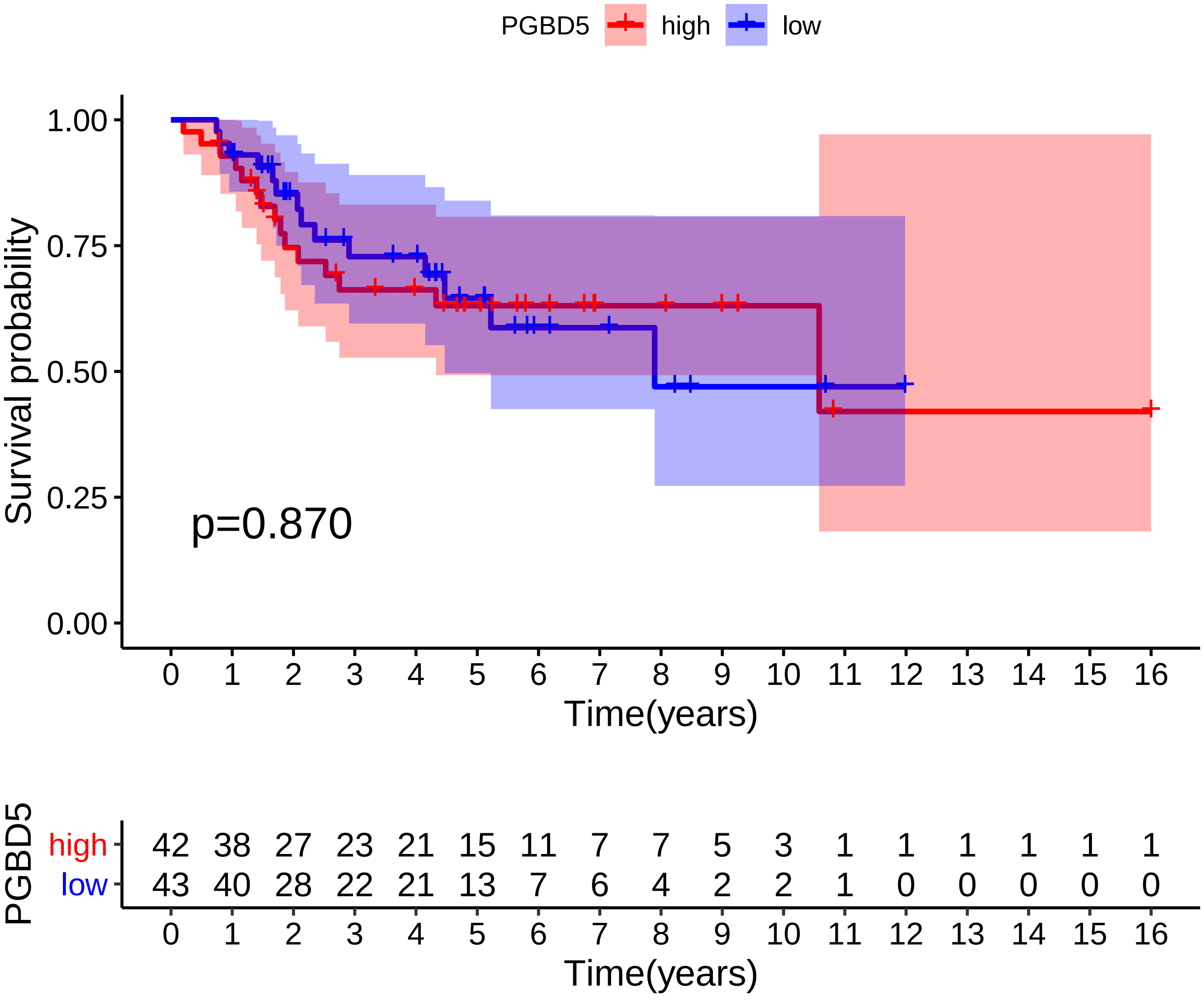

PGBD5
high
low
1.00
0.75
0.50
0.25
0.00
Survival probability
p=0.870
0
1
2
3
4
5
6
7
8
9
10
11
12
13
14
15
16
Time(years)
PGBD5
high
low
42 38 27 23 21 15 11 7 7 5 3 1 1 1 1 1 1
43 40 28 22 21 13 7 6 4 2 2 1 0 0 0 0 0
Time(years)
